# QiShenYiQi pills, a Chinese patent medicine, increase bioavailability of atorvastatin by inhibiting Mrp2 expression in rats

**DOI:** 10.1080/13880209.2021.2021949

**Published:** 2022-01-08

**Authors:** Congyang Ding, Yajing Li, Xiao Li, Lu Meng, Ran Fu, Xiaonan Wang, Ying Li, Yinling Ma, Zhanjun Dong

**Affiliations:** a National Clinical Drug Monitoring Center, Hebei General Hospital, Shijiazhuang, China; b Department of Pharmacy, The Second Hospital of Shijiazhuang, Shijiazhuang, China; c Graduate School, Hebei Medical University, Shijiazhuang, China

**Keywords:** Metabolic enzymes, transporters, drug-drug interaction

## Abstract

**Context:**

Atorvastatin (ATV) and QiShenYiQi pills (QSYQ), a Chinese patent medicine, are often co-prescribed to Chinese cardiovascular patients. The effects of QSYQ on the pharmacokinetics of ATV have not been studied.

**Objective:**

We investigated the influence of QSYQ on the pharmacokinetics of ATV and its metabolites upon oral or intravenous administration of ATV to rats.

**Materials and methods:**

Sprague-Dawley rats (*n* = 5/group) were pre-treated with oral QSYQ (675 mg/kg) or vehicle control for 7 days and then orally administrated ATV (10 mg/kg) or intravenously administrated ATV (2 mg/kg). Serum concentrations of ATV and metabolites were determined by ultra-high performance liquid chromatography tandem mass spectrometry. Expression of metabolic enzymes and transporters in jejunum and ileum were measured by quantitative real-time PCR and Western blot.

**Results:**

QSYQ resulted in an increase of AUC_0-12 h_ of ATV from 226.67 ± 42.11 to 408.70 ± 161.75 ng/mL/h and of *C_max_* of ATV from 101.46 ± 26.18 to 198.00 ± 51.69 ng/mL and in an increased of *para-*hydroxy atorvastatin from 9.07 ± 6.20 to 23.10 ± 8.70 ng/mL in rats administered ATV orally. No change was observed in rats treated intravenously. The expression of multidrug resistance-associated protein 2 mRNA and protein decreased in ileum, and the mRNA of P-glycoprotein decreased in jejunum, though no change in protein expression was found.

**Discussion and conclusions:**

QSYQ increased bioavailability of ATV administered orally through inhibiting the expression of Mrp2 in ileum. Clinicians should pay close attention to potential drug-drug interactions between ATV and QSYQ.

## Introduction

Coronary heart disease (CHD) is caused by myocardial ischaemia, hypoxia or necrosis due to the luminal narrowing or occlusion that results from atherosclerosis of coronary arteries. In 2019, a report on cardiovascular diseases in China demonstrated that the number of patients with CHD had increased to eleven million (Hu et al. 2019).

Atorvastatin (ATV) is commonly used for the secondary prevention of CHD. It is an inhibitor of 3-hydroxy-3-methylglutaryl coenzyme A reductase and thus lowers endogenous cholesterol synthesis (Hirota and Ieiri 2015). ATV is administrated orally, and it is absorbed by organic anion transporting polypeptides 2B1 (OATP2B1/Oatp2b1) and excreted into the intestinal lumen by P-glycoprotein, breast cancer resistance protein (BCRP/Bcrp) and multidrug resistance-associated protein 2 (MRP2/Mrp2). This process results in a reduction of bioavailability (Dong et al. 2008; Shin et al. 2017; Brackman and Giacomini 2018).

ATV is taken up into hapatocytes by organic anion transporting polypeptides 1B1 and 1B3 in human or by organic anion transporting polypeptides 1b2 (Oatp1b2) in rats. The major route of metabolism of ATV involves oxidation to *ortho*-hydroxy atorvastatin (*o*-ATV) and *para*-hydroxy atorvastatin (*p*-ATV) by CYP3A4 in human or by Cyp3A1 in rats. These metabolites are the key active forms of ATV and play important role in lowering cholesterol (Tamai 2012; Chang et al. 2014; Zhang 2015; Drozdzik et al. 2019). A serious potential adverse reaction of ATV treatment is rhabdomyolysis, which causes proteinuria leading to acute renal failure. Therefore, clinicians need to monitor patients for the adverse effects, especially when co-administrating drugs that might affect the absorption, distribution, metabolism and excretion (ADME) of ATV, such as rifampin, ketoconazole and metformin (Chang et al. 2014; Shin et al. 2017).

ATV has long been co-prescribed with a Chinese patent medicine, QiShenYiQi pills (QSYQ), which are formulated from extracts of *Astragalus membranaceus* Bunge (Leguminosae)*, Salvia miltiorrhizae* C. Y.Wu (Lamiaceae)*, Panax notoginseng* (Burkill) F.H.Chen (Araliaceae), and *Dalbergia odorifera* T.C.Chen (Fabaceae). QSYQ have been shown to increase coronary blood flow, attenuate energy metabolism deficiencies, protect vascular endothelial cells from inflammatory factors and inhibit local inflammatory response to stabilise atherosclerotic plaques (Cui et al. 2018). There are many active ingredients in QSYQ, including astragaloside IV, danshensu, salvianolic acid B, tanshinone IIA, tanjin, and *Panax notoginseng* saponins. Previous studies have shown that astragaloside IV affects the expression of CYP3A4 and P-glycoprotein (Zhang et al. 2016), tanshinone IIA induces CYP3A4 enzyme activity (Yu et al. 2009), and salvianolic acid B inhibits CYP3A4 enzyme activity (Wang et al. 2011). Tanshinone IIA and tanjin also are known to inhibit the activity of P-glycoprotein, BCRP, and OATP isoforms to different degrees (Li and Lai 2017; Yang et al. 2019). *Panax notoginseng* saponins were not found to have effect on metabolic enzymes and transporters, but metabolites generated by intestinal flora exhibited varying degrees of inhibition on P-glycoprotein. QSYQ contain these active ingredients and thus may impact the metabolism of other drugs, but the effects of this formulation on metabolic enzymes and transporters have not yet been investigated.

ATV and QSYQ have been prescribed together to tens of thousands of Chinese CHD patients, and these patients tend to be treated with these two formulations for extended periods of time. Therefore, it is possible that the adverse reactions that accompany ATV treatment, especially rhabdomyolysis, may be exacerbated by slowed ATV metabolism caused by drug-drug interactions (Khalilieh et al. 2017). Accordingly, whether the pharmacokinetics of ATV are affected by co-treatment with QSYQ needs to be explored. Considering that *o*-ATV and *p*-ATV are active metabolites produced from ATV by CYP3A4 or Cyp3A1, we set out to detect the impact of QSYQ on concentration of *o*-ATV and *p*-ATV, which we propose are reflective of changes to the activities of the metabolic enzymes.

In this study, we treated rats with QSYQ or vehicle control and compared serum concentration of ATV, *o*-ATV and *p*-ATV by ultra-high performance liquid chromatography tandem mass spectrometry (UPLC-MS/MS) analysis. In addition, the expression of mRNA coding for Cyp3A1, Cyp3A2, Oatp1b2 in liver and P-glycoprotein, Oatp2b1, Bcrp and Mrp2 in jejunum and ileum were measured by quantitative real-time PCR (qRT-PCR) and the levels of expression of P-glycoprotein in jejunum and Mrp2 in the ileum were measured by Western blotting (WB). Our analyses suggest that QSYQ slows the metabolism of ATV and may have adverse impacts on CHD patients.

## Materials and methods

### Chemicals and reagents

ATV (purity ≥ 98%) was purchased from Solarbio Science & Technology (Beijing, China). *o*-ATV (purity ≥ 96%), *p*-ATV (purity ≥ 95%) and simvastatin acid (SVA, purity ≥ 92%), which was used as an internal standard, were purchased from Toronto Research Chemicals (Toronto, Canada). QSYQ (batch number: 20181201) were purchased from Tasly Pharmaceutical (Tianjin, China). Saline solution was purchased from Shijiazhuang No.4 Pharmaceutical (Shijiazhuang, China). Polyethylene glycol 400 and carboxymethylcellulose sodium were purchased from Sangon Biotech (Shanghai, China). HPLC-grade methanol, acetonitrile and formic acid were purchased from Fisher Scientific (Pittsburgh, PA, USA). Ultrapure water was purchased from Wahaha Group (Hangzhou, China). RNAsimple Total RNA Kit, FastQuant RT Kit (with gDNase), and SuperReal PreMix Plus (SYBR Green) were purchased from Tiangen Biotech (Beijing, China). The rabbit anti-P-glycoprotein (AF5185), rabbit anti-Mrp2 (DF3873), rabbit anti-ATP1A1 (AF6109) antibodies and goat anti-rabbit secondary antibody (S0001) were purchased from Affinity Biosciences (Cincinnati, OH, USA).

### Animal experiments

Sprague-Dawley rats (male, 200–250 g) were purchased from the Experimental Animals Centre of Hebei Medical University (Shijiazhuang, China). Rats were housed in a room that was temperature controlled at 23–27 °C, and the humidity was kept in the range of 40–70%. A 12 h light-dark cycle was maintained. Rats had free access to food and water and were adapted to the environment for seven days prior to experiments. All animal experiments were in accordance with the Public Health Service Policy on Humane Care and Use of Laboratory Animals (National Institutes of Health Publications No. 15-8013, revised 2015) and animal experimental protocols were approved by the Ethics Committee of Hebei General Hospital.

### *In vivo* pharmacokinetic studies

Twenty rats were randomly and evenly divided into four groups: ATV administrated orally without QSYQ (Group I), ATV administrated orally with QSYQ (Group II); ATV administrated intravenously without QSYQ (Group III); and ATV administrated intravenously with QSYQ (Group IV). ATV to be administrated orally was dissolved in 0.5% carboxymethylcellulose sodium and administrated at a dose five-fold higher than the recommended human dose of 10 mg/kg. ATV to be administrated intravenously were dissolved in polyethylene glycol 400/saline (1:3) to achieve a human equivalent oral dose of 2 mg/kg. QSYQ was dissolved in saline and administered at a concentration of 675 mg/kg, five-fold higher than the human recommended dose. Saline or QSYQ was given through the gastrointestinal tract once daily for seven consecutive days. On the eighth day of the experiment, after 12 h with no food but free access to water, rats received oral or intravenous ATV 1 h after saline or QSYQ. Approximately 0.2–0.3 mL of blood was collected from the orbital venous plexus with a heparinised capillary tube, and blood samples were placed in heparinised tubes at 0, 0.083, 0.167, 0.25, 0.333, 0.5, 0.75, 1, 1.5, 2, 4, 6, 12 h for Group I and Group II, and 0, 0.033, 0.083, 0.167, 0.333, 0.5, 1, 1.5, 2, 4, 6 h for Group III and Group IV. The rats were provided 2 mL water after 2 h to offset blood loss, and they were allowed free access to water and food after 4 h. All blood samples were immediately centrifuged at 3000 rpm for 10 min at 4 °C, and plasma was collected and stored in a −80 °C freezer prior to UPLC-MS/MS analysis.

### Sample analysis

**Figure 1.**
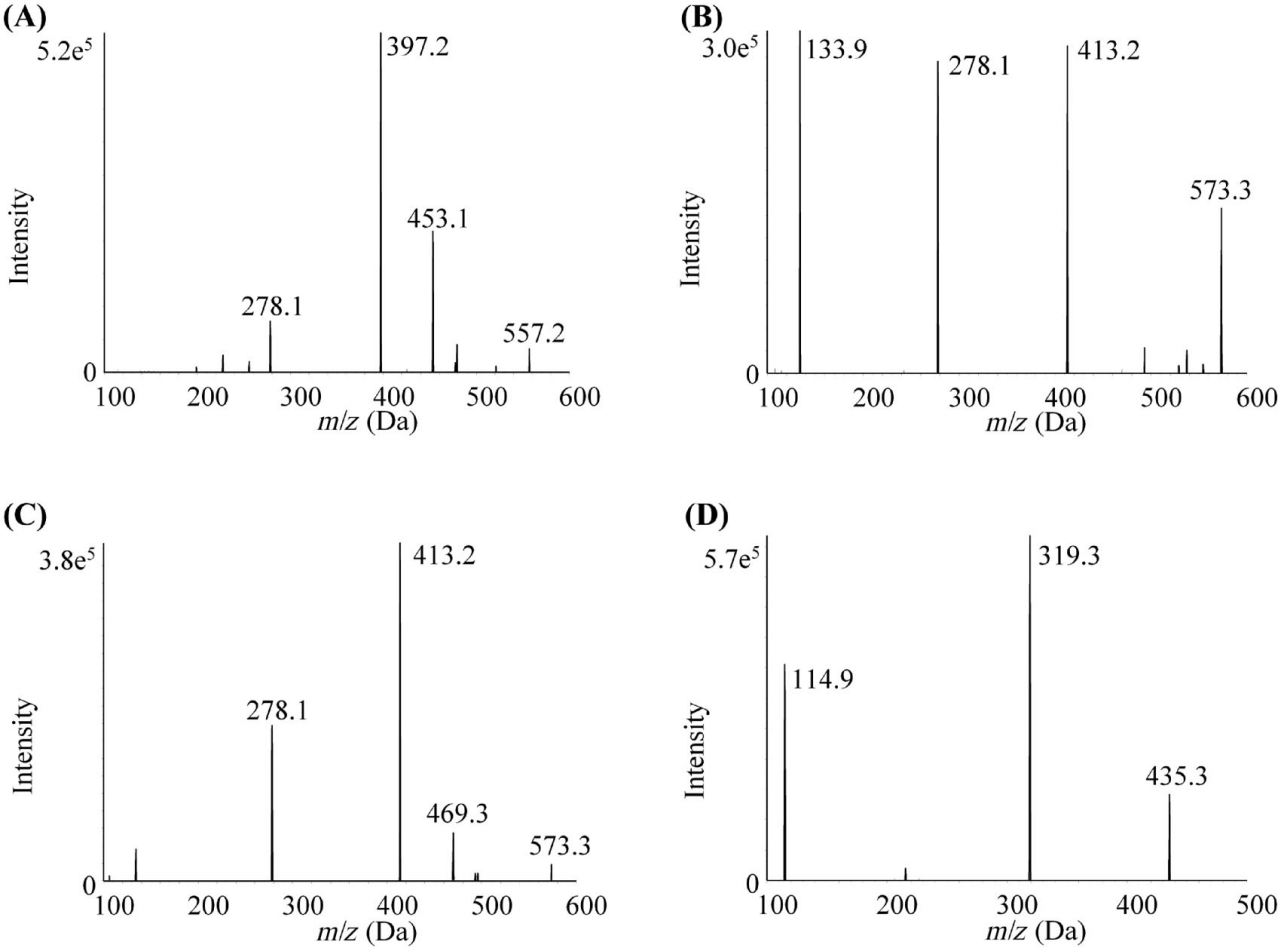
The product ion mass spectra of ATV, *o*-ATV, *p*-ATV and SVA. (A) ATV; (B) *o*-ATV; (C) *p*-ATV; (D) SVA.

ATV and its two active metabolites *o*-ATV and *p*-ATV were quantitatively determined by a validated UPLC-MS/MS method. The UPLC-MS/MS system includes an AB Sciex 5500 triple quadrupole tandem mass spectrometer equipped with an electrospray ionisation interface (Framingham, MA, USA). Negative ionisation mode and multiple reaction monitoring mode were selected for quantification with a C18 column (Waters XBridge BEH C18 column, 2.1 × 100 mm, 2.5 μm). The gradient of the mobile phase consisting of 0.1% formic acid in water (A) and acetonitrile (B) with a flow rate of 0.25 mL/min was set as follows: 0–0.1 min, 50% B; 0.1–2 min 50–70%, B; and 2–5 min, 70%, B. The pre-column equilibration time and sample injection volume were 1 min and 5 μL. The parameters of mass spectrometry for each analyte are shown in Table 1 and product ion mass spectra are shown in Figure 1.

**Table 1.** Experimental setting of the tandem mass spectrometry for ATV, *o*-ATV, *p*-ATV and SVA.

Experimental setting	ATV	*o*-ATV	*p*-ATV	SVA
Ion pairs	557.2→278.1	573.3→278.1	573.3→278.1	435.3→319.3
Ion source temperature (°C)	450	450	450	450
Ion spray voltage (V)	−4500	−4500	−4500	−4500
Declustering Potential (V)	−100	−75	−85	−80
Collision Energy (eV)	−60	−56	−58	−22

ATV: atorvastatin; *o*-ATV: *ortho*-hydroxy atorvastatin; *p*-ATV: *para*-hydroxy atorvastatin; SVA: simvastatin acid.

Sample preparation was performed by liquid-liquid extraction on ice. Aliquots of 50 μL of plasma and 5 μL of internal standard working solution were place in a 1.5 mL centrifuge tube and vortexed for 1 min. Then 50 μL water with formic acid, pH 4.5, was added and the sample was vortexed again for 30 s. Ethyl acetate (200 μL) was added and the mixture was vortexed for 1 min and centrifuged for 10 min at 4 °C. A 160 μL portion of supernatant was transferred to a new 1.5 mL centrifuge tube and evaporated to dryness under a gentle stream of nitrogen gas at 40 °C. The residue was reconstituted in 100 µL of mobile phase, vortexed for 1 min and centrifuged for 10 min at 4 °C after vortexing for 1 min. Then, 60 µL of the supernatant was pipetted into a UPLC sample vial, and 5 µL of the sample was injected for UPLC-MS/MS analysis.

The limits of detection, defined as a signal-to-noise ratio of 3, were 0.02 ng/mL for ATV and 0.1 ng/mL for both *o*-ATV and *p*-ATV. The limits of quantification based on a signal-to-noise ratio of 10, were 0.1 ng/mL for ATV and 0.5 ng/mL for both *o*-ATV and *p*-ATV. Linear calibration curves for ATV, *o*-ATV and *p*-ATV were constructed in blank matrix at concentrations of 0.5, 2.5, 7.5, 15, 25, 75, 150, and 250 ng/mL. The quality control (QC) samples were prepared at low (1 ng/mL), medium (50 ng/mL) and high (200 ng/mL) concentrations in the same way as plasma sample for calibration. The intra-day, inter-day, matrix effect, extraction recovery and dilution integrity precision and accuracy of the QC samples for each analyte were less than 15%. The short-term stability (at room temperature for 2 h), long-term stability (at −80 °C for 7 days), freeze-thaw stability (−80 °C to room temperature for three cycles) and post-preparative stability (the extracts on the bench top at room temperature for 8 h) of the QC samples were determined, and precision and accuracy were less than 15% under all conditions.

### mRNA expression analysis by qRT-PCR

After the last blood samples were drawn, all rats were killed by cervical dislocation. Samples of liver, jejunum and ileum were quickly removed, perfused with ice-cold saline and stored at −80 °C. Total mRNA was extracted using an RNAsimple Total RNA Kit according the manufacturer’s instructions. The quality and purity of the mRNA were determined by UV spectrophotometry at 260 nm and 280 nm. Then, 2 μg of total RNA was added for the first chain of cDNA synthesis with FastQuant RT Kit, and the cDNA was used for subsequent PCR reactions. The reaction volume used for PCR was 20 μL. The amplification steps were: 15 min with initial denaturation at 95 °C to fully activate the hot start enzyme, followed by denaturation at 95 °C for 30 s and annealing at 60 °C for 32 s. A total of 40 cycles were carried out. *β-*Actin was used as the internal reference gene. The relative expression levels of mRNA were calculated by 2^−ΔΔCT^ method relative to *β-Actin* as the internal reference gene. The gene corresponding to the analysed mRNA sequences were Cyp3A1/*Cyp3A1*, Cyp3A2/*Cyp3A2,* Oatp1b2/*Slco1b2* in liver, and P-glycoprotein/*Abcb1a*, Oatp2b1/*Slco2b1*, Bcrp/*Abcg2*, Mrp2/*Abcc2* in jejunum and ileum. Primer sequences are shown in Table 2.

**Table 2.** The mRNA primer sequences.

Genes	Forward	Reverse
*Cyp3A1*	TGCATTGGCATGAGGTTTGC	TTCAGCAGAACTCCTTGAGGG
*Cyp3A2*	TTCTAAAGGTTCTGCCACGGG	CCATCACAGACCTTGCCAACT
*Slco1b2*	GCTGATTGGAATTGGCTGCTT	GGTGAAGGTCCAGTGGGTGA
*Abcb1a*	TCTGGTATGGGACTTCCTTGGT	TCCTTGTATGTTGTCGGGTTTG
*Slco2b1*	TGCGAGTGTTGGCCTGGAT	TCATGGTCATAGTAGCGGCAGA
*Abcg2*	TGAAGAGTGGCTTTCTAGTCCG	TTGAAATTGGCAGGTTGAGGTG
*Abcc2*	ATGCGGCGTATTCCAGTTTC	TTGCTGGTGACTGACCTTGTTT
*β-actin*	TGCTATGTTGCCCTAGACTTCG	GTTGGCATAGAGGTCTTTACGG

### Protein expression analysis by WB

The protein levels of P-glycoprotein in jejunum and Mrp2 in ileum were measured by WB. Protein levels were normalised to the quantity of ATP1A1. Proteins were isolated from tissue samples with a Membrane and Cytosol Protein Extraction Kit. Protein samples (50 μg total protein) were loaded onto 8% sodium dodecyl sulfate-polyacrylamide gel and transferred onto polyvinylidene fluoride membranes. The membranes were blocked with Tris-buffered saline containing 0.1% Tween 20 (TBST) solution containing 5% skim milk powder for 2 h and washed. Then, membranes were incubated with primary antibodies against P-glycoprotein, Mrp2 or ATP1A1 at dilution of 1:1000 in TBST solution at 4 °C overnight. On the second day, the membranes were washed three times with TBST solution, and then incubated with secondary antibody diluted (1/5000 in TBST) for 1 h, and washed again three times with TBST. Proteins on membranes were visualised by enhanced chemiluminescence and detected with a MiniChemi 610 PlusChemiluminescence gel imaging system (Beijing Sage Creation Science Co., LTD, Beijing, China). The densities of protein bands were quantified by ImageJ 1.53 software (National Institutes of Health, Bethesda, MD, USA).

### Statistical analysis

The pharmacokinetic parameters were calculated with DAS 2.1.1 software (Mathematical Pharmacology Professional Committee of China, Shanghai, China). Pharmacology, mRNA and protein data were analysed by SPSS 21.0 software (SPSS Inc., La Jolla, CA, USA), and *t*-tests and nonparametric rank-sum test were applied to compare Group I with Group II and Group III with Group IV. The data were expressed as mean ± SD and value of *p* < 0.05 was considered to indicate statistically significance.

## Results

### Pharmacokinetics of ATV, o-ATV, p-ATV

**Figure 2.**
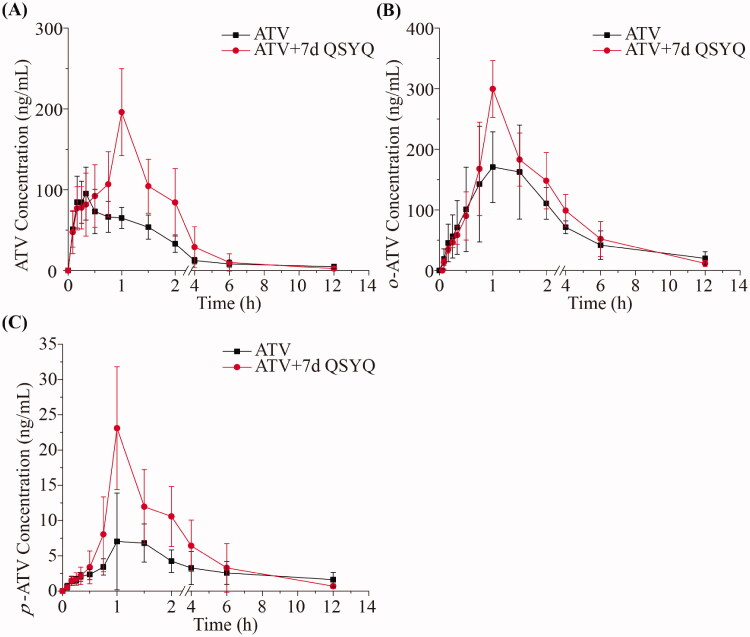
The pharmacokinetic profiles of ATV, *o*-ATV and *p*-ATV after oral administration of ATV without or with QSYQ in rats. (A) ATV; (B) *o*-ATV; (C) *p*-ATV. Data are shown as mean ± SD (*n* = 5). *p < 0.05, **p < 0.01 compared to without QSYQ.

**Figure 3.**
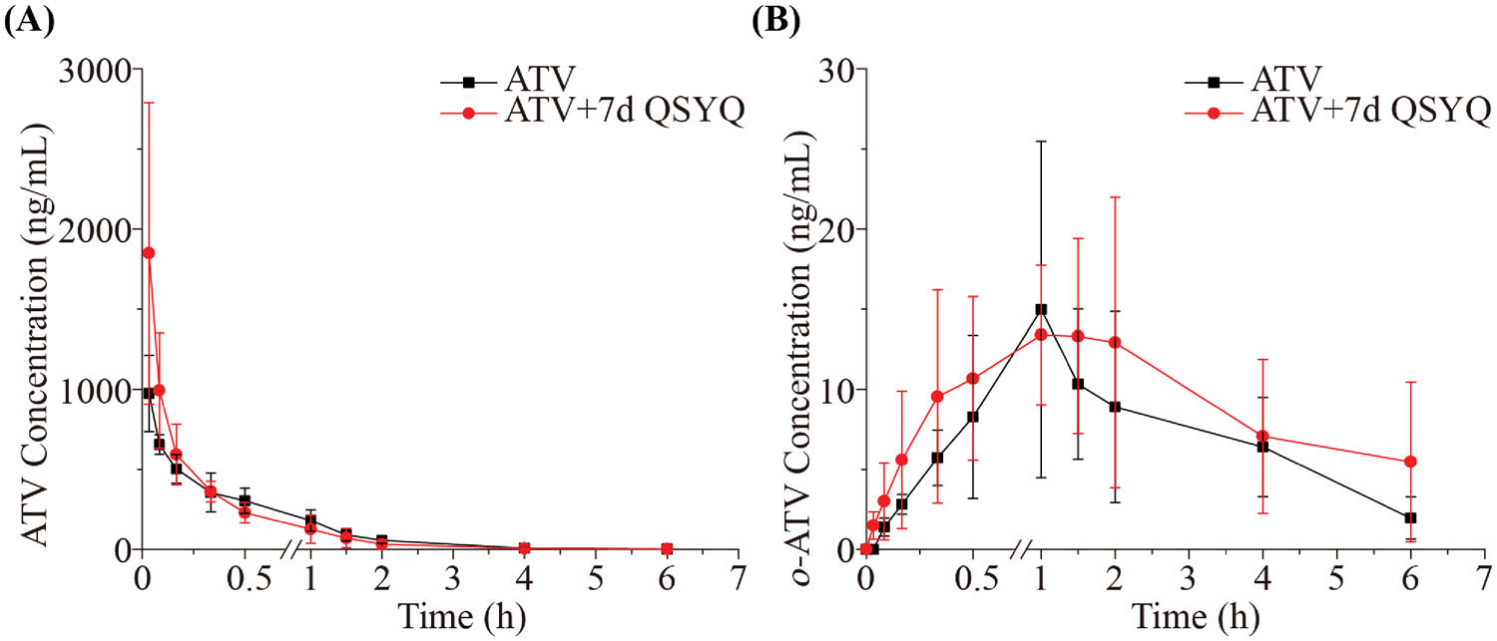
The pharmacokinetic profiles of ATV and *o*-ATV after intravenous administration of ATV without or with QSYQ in rats. (A) ATV; (B) *o*-ATV. Data are shown as mean ± SD (*n* = 5).

In this study, we employed a validated method to determine concentration of ATV, *o*-ATV and *p*-ATV. Pharmacokinetic parameters and the variations of the mean plasma concentration-time curves in groups that were administered ATV orally (Group I and Group II) are shown in Table 3 and Figure 2. Pharmacokinetic parameters and the variations of the mean plasma concentration-time curves in groups that were administered ATV intravenously (Group III and Group IV) are shown in Table 4 and Figure 3. When QSYQ was co-administered to rats treated with ATV via the oral route (compare Group I and II), the area under the plasma concentration-time curve from 0 to 12 h (AUC_0–12 h_) of ATV increased from 226.67 ± 42.11 to 408.70 ± 161.75 ng/mL/h (*p* < 0.05). Similarly, the maximum serum concentrations (*C_max_*) of ATV and *p*-ATV increased significantly (101.46 ± 26.18 *vs.* 198.00 ± 51.69 ng/mL and 9.07 ± 6.20 *vs.* 23.10 ± 8.70 ng/mL, respectively), and the serum half-life (*t_1/2_*) of ATV and *p*-ATV decreased significantly (5.47 ± 3.46 *vs.* 1.40 ± 0.41 h and 9.05 ± 3.31 *vs.* 2.17 ± 0.74 h, respectively), The volume of distribution (V_z_) of ATV and *p*-ATV decreased (270.51 ± 119.65 *vs.* 54.71 ± 26.62 L/kg, 2814.17 ± 1409.11 *vs.* 601.61 ± 378.37 L/kg, respectively) upon treatment with QSYQ, and differences were significant (*p* < 0.05). The results showed no significant differences in pharmacokinetic parameters of *o*-ATV (*p* > 0.05).

**Table 3.** The pharmacokinetic parameters of ATV, *o*-ATV and *p*-ATV in rat plasma after oral administration of ATV with or without QSYQ.

Oral administration PK parameters	ATV		*o*-ATV		*p*-ATV	
	Without QSYQ	With QSYQ	Without QSYQ	With QSYQ	Without QSYQ	With QSYQ
AUC_0–12 h_ (ng/mL/h)	226.67 ± 42.11	408.70 ± 161.75*	729.47 ± 150.54	906.14 ± 145.34	34.91 ± 18.77	59.24 ± 25.12
AUC_0–∞_ (ng/mL/h)	273.36 ± 93.26	410.97 ± 164.13	865.33 ± 229.23	949.09 ± 133.93	56.26 ± 32.69	60.91 ± 25.78
*t_1/2_* (h)	5.47 ± 3.46	1.40 ± 0.41*	4.15 ± 1.99	2.56 ± 0.98	9.05 ± 3.31	2.17 ± 0.74**
*T_max_* (h)	0.43 ± 0.33	0.95 ± 0.11	1.05 ± 0.27	1	1.25 ± 0.35	1
V_z_ (L/kg)	270.51 ± 119.65	54.71 ± 26.62*	68.79 ± 23.90	40.25 ± 18.91	2814.17 ± 1409.11	601.61 ± 378.37*
CL_z_ (L/h/kg)	39.64 ± 11.63	27.02 ± 8.72	12.22 ± 3.21	10.69 ± 1.41	235.70 ± 142.44	187.55 ± 69.46
*C_max_* (ng/mL)	101.46 ± 26.18	198.00 ± 51.69**	203.06 ± 84.97	299.60 ± 46.70	9.07 ± 6.20	23.10 ± 8.70*

Data are shown as mean ± SD (*n* = 5).

ATV: atorvastatin; *o*-ATV: *ortho*-hydroxy atorvastatin; *p*-ATV: *para*-hydroxy atorvastatin; QSYQ: QiShenYiQi pills.

*p < 0.05, **p < 0.01 compared to without QSYQ.

**Table 4.** The pharmacokinetic parameters of ATV and *o*-ATV in rat plasma after intravenous administration of ATV with or without QSYQ.

Intravenous administration PK parameter	ATV		*o*-ATV	
	Without QSYQ	With QSYQ	Without QSYQ	With QSYQ
AUC_0–6 h_ (ng/mL/h)	549.50 ± 99.25	548.92 ± 90.01	42.66 ± 19.65	55.21 ± 28.56
AUC_0–∞_ (ng/mL/h)	550.62 ± 99.57	549.99 ± 90.71	50.01 ± 20.42	85.52 ± 75.55
*t_1/2_* (h)	0.71 ± 0.90	0.74 ± 0.12	2.09 ± 0.88	3.04 ± 1.79
V_z_ (L/kg)	3.83 ± 1.12	3.90 ± 0.34	138.97 ± 67.25	118.41 ± 45.57
CL_z_ (L/h/kg)	3.73 ± 0.70	3.73 ± 0.73	47.06 ± 22.27	32.97 ± 13.85

ATV: atorvastatin; *o*-ATV: *ortho*-hydroxy atorvastatin; QSYQ: QiShenYiQi pills.

Data are shown as mean ± SD (*n* = 5).

**Figure 4.**
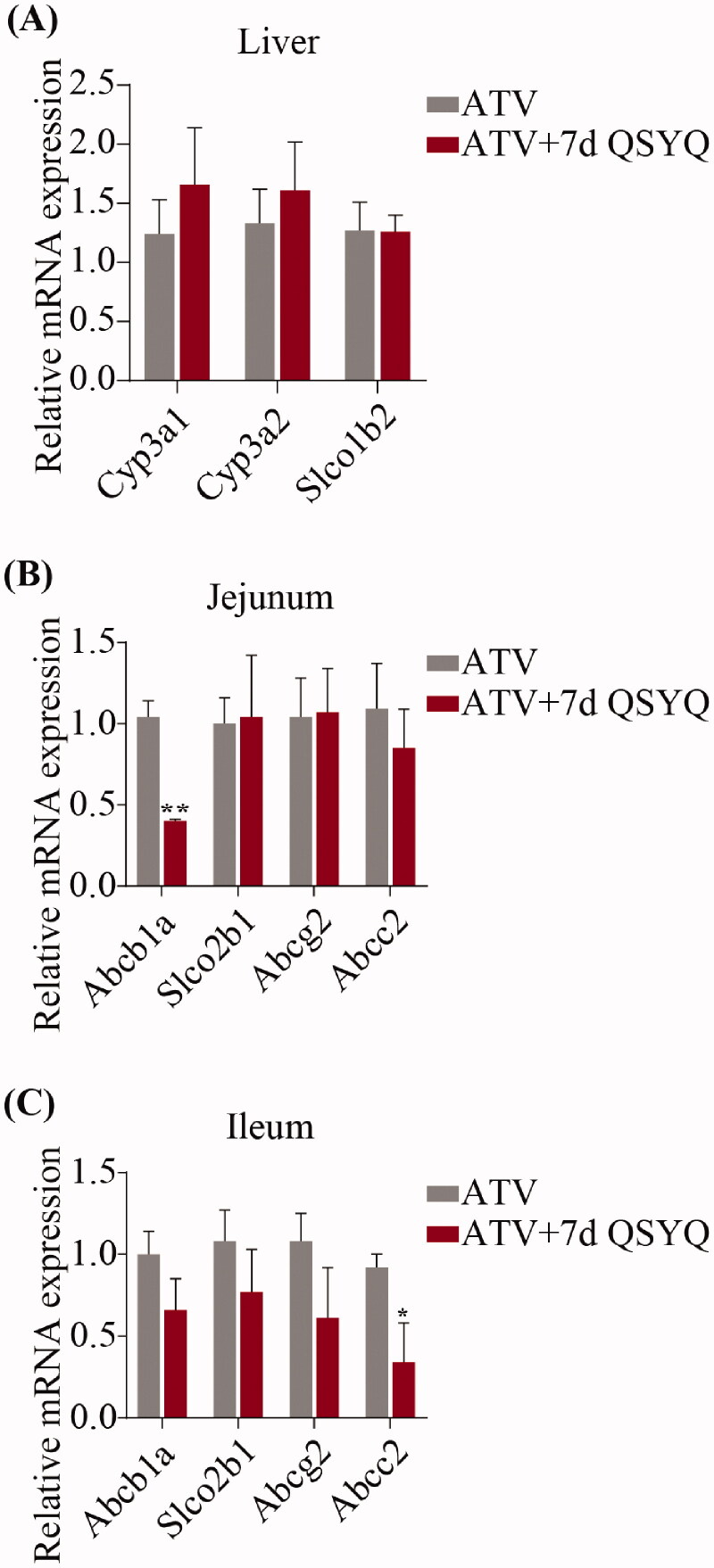
Relative mRNA expression of *Cyp3A1*, *Cyp3A2*, *Slco1b2* in liver and *Abcb1a*, *Slco2b1*, *Abcg2*, *Abcc2* in jejunum and ileum after oral administration of ATV without or with QSYQ. (A) Relative mRNA expression of *Cyp3A1*, *Cyp3A2*, *Slco1b2* in liver; (B) Relative mRNA expression of *Abcb1a*, *Slco2b1*, *Abcg2*, *Abcc2* in jejunum; (C) Relative mRNA expression of *Abcb1a*, *Slco2b1*, *Abcg2*, *Abcc2* in ileum. Data are shown as mean ± SD (*n* = 3). *p < 0.05, **p < 0.01 compared to without QSYQ.

On the other hand, in rats administered ATV intravenously, we did not observe any significant changes in the pharmacokinetic parameters of ATV or *o*-ATV (*p* > 0.05) upon co-treatment with QSYQ (compare Groups III and IV, Table 3 and Figure 4). In these groups, the concentration of *p*-ATV was not determined in most cases because it was below the lower limit of quantification. Together, these data suggest that QSYQ significantly increases plasma exposure of ATV when the drug is administered orally but that no observable change occurs when ATV is administered intravenously.

### mRNA expression

**Figure 5.**
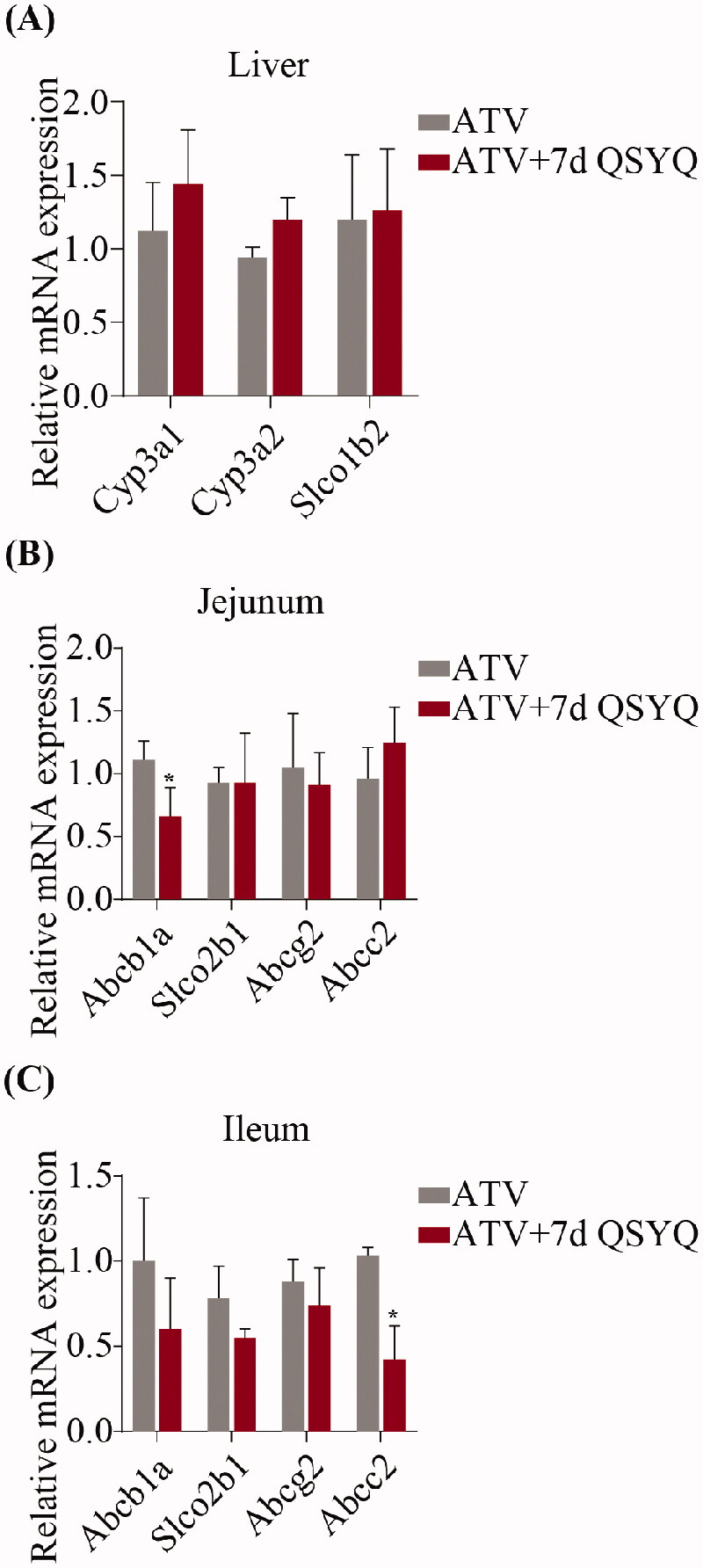
Relative mRNA expression of *Cyp3A1*, *Cyp3A2*, *Slco1b2* in liver and *Abcb1a*, *Slco2b1*, *Abcg2*, *Abcc2* in jejunum and ileum after intravenous administration of ATV without or with QSYQ. (A) Relative mRNA expression of *Cyp3A1*, *Cyp3A2*, *Slco1b2* in liver; (B) Relative mRNA expression of *Abcb1a*, *Slco2b1*, *Abcg2*, *Abcc2* in jejunum; (C) Relative mRNA expression of *Abcb1a*, *Slco2b1*, *Abcg2*, *Abcc2* in ileum. Data are shown as mean ± SD (*n* = 3). *p < 0.05 compared to without QSYQ.

In consideration of the transport of ATV by several intestinal transporters and its biotransformation by specific liver enzyme, the expression of *Cyp3A1*, *Cyp3A2* and *Slco1b2* in liver and *Abcb1a*, *Slco2b1*, *Abcg2* and *Abcc2* in jejunum and ileum were detected by qRT-PCR (Figures 4 and 5). In the groups treated with ATV orally, expression of *Abcb1a* decreased (*p* < 0.05) in jejunum and expression of *Abcc2* decreased (*p* < 0.05) in ileum upon treatment with QSYQ (compare Groups I and II; Figure 4). Similarly, in the groups treated with ATV intravenously, QSYQ treatment correlated with a significant decrease in the expression of *Abcb1a* (*p* < 0.05) in jejunum and a significant decrease in the expression of *Abcc2* (*p* < 0.05) in ileum. QSYQ treatment led to increased expression of *Cyp3A1* and *Cyp3A2* in the liver and decreased expression of *Abcb1a*, *Slco2b1* and *Abcg2* in ileum, although these differences did not rise to the level of statistical significance. There were no significant changes detected in the expression of mRNA of other analysed genes upon administration of QSYQ (*p* > 0.05).

### Protein expression

**Figure 6.**
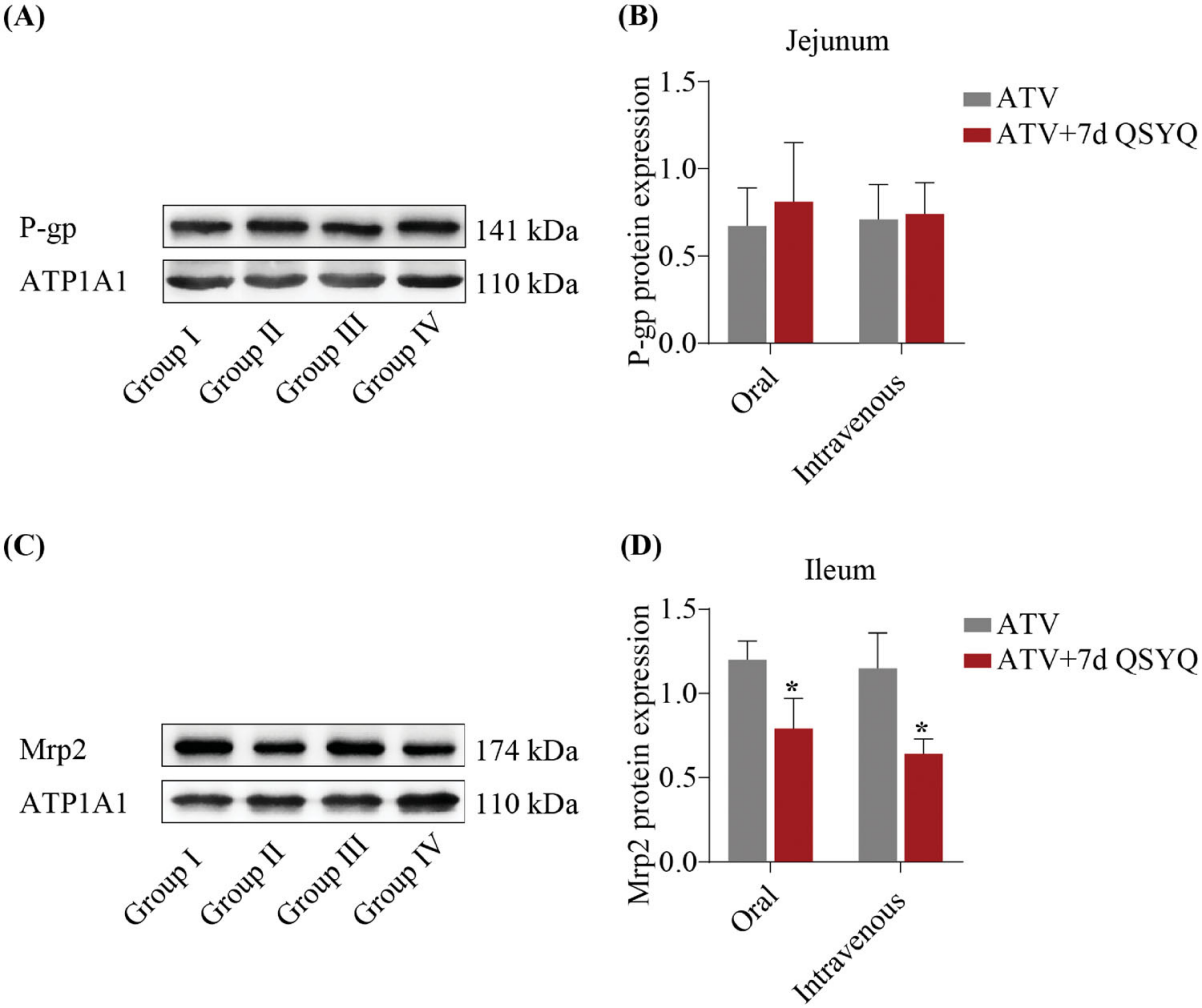
Protein expression of P-glycoprotein in jejunum and Mrp2 in ileum after oral or intravenous administration of ATV without or with QSYQ. (A) and (B) Protein expression of P-glycoprotein in jejunum; (C) and (D) Protein expression of Mrp2 in ileum. Data are shown as mean ± SD (*n* = 3). *p < 0.05 compared to without QSYQ.

To further investigate QSYQ-mediated changes in expression, the protein expression of P-glycoprotein in the jejunum and Mrp2 in the ileum were detected by western blots and expression levels were quantified, as shown in Figure 6. Upon QSYQ treatment of rats treated orally with ATV, the expression of the Mrp2 protein decreased (*p* < 0.05) (compare Groups I and II). In the intravenously treated rats, QSYQ resulted in a decrease of Mrp2 protein expression (*p* < 0.05) (compare Groups III and IV). The level of P-glycoprotein was not significantly changed under any condition (*p* > 0.05).

## Discussion

ATV is a potent drug in the lowering of low-density lipoprotein level and the stabilising of plaques in the management of cardiovascular disease (Reiner 2014). Adverse reactions, including myopathy and rhabdomyolysis, are associated with systemic statin exposure, and these reactions may be especially important when co-administered with drugs impacting systemic ATV exposure (Chang et al. 2014; Shin et al. 2017). ATV is prescribed to patients with hyperlipidaemia or CHD, and these patients frequently take patent Chinese medicines in hope of augmenting the cardioprotective effects.

QSYQ represent one such example of a patent Chinese medicine that are frequently taken with ATV. QSYQ contains many active ingredients as extracted via modern pharmaceutical technologies, these ingredients include extracts of *Astragalus membranaceus*, *Salvia miltiorrhizae*, *Panax notoginseng*, and *Dalbergia odorifera* (Lv et al. 2017). The complex nature of QSYQ suggests that this formulation might potentially impact the ADME of ATV, yet such interactions had not been studied previously. In this study, then, we analysed the impact of QSYQ on the pharmacokinetics of ATV, *o*-ATV and *p*-ATV in rats administered ATV via oral and intravenous routes. In addition, because ATV is known to be transported by P-glycoprotein, Oatp2b1, Bcrp and Mrp2 on intestinal epithelial cells and Oatp1b2 on hepatocyte and to be transformed to the major metabolite *o*-ATV and minor metabolite *p*-ATV by Cyp3A1 in liver (Morse et al. 2019), we also analysed expression of these gene products. Notably, because studies have shown that the promoter region of *Cyp3A1* is more similar to that of *CYP3A4* than *Cyp3A2*, many other researchers have only examined the *Cyp3A1* subtype, however, we investigated both *Cyp3A1* and *Cyp3A2* due to their 88% identity (Takada et al. 2004; Bao et al. 2012; Handa et al. 2013).

The recommended dose of ATV is between 10 and 80 mg once per day, and the preferred dose among Chinese is 20 mg (Hua et al. 2018; Yang et al. 2018). This dose was converted to an appropriate dose in rats according to body surface area. The equation used for this purpose was rat dose = human daily dose × rat conversion factor/rat weight, the rat conversion factor used was 0.018, considering average masses of 70 kg for human and 0.2 kg for rat (Wang and Feng 2019; Li et al. 2020). The results of this equation were rounded up to a dose of 2 mg/kg bodyweight. However, we administered 10 mg/kg doses to rats in the oral administration groups. While this dose is 5-fold higher than the rat conversion dose, it is consistent with doses used in previous studies (Dong et al. 2008; Vats et al. 2012; Malekinejad et al. 2014). The dose administrated intravenously was 2 mg/kg, which is also consistent with a previous study (Shu et al. 2016). Similarly, the dose of QSYQ converted from clinical use is approximately 135 mg/kg, according to the recommended dose of 1.5 g per day for human patients and relative body surface area of human and rats (Shang et al. 2013). In our study, the dose of QSYQ administrated to rats was increased to 675 mg/kg, which is similarly 5-fold higher than the rat conversion dose, in order to better observe any potential drug-drug interactions.

Considering that ADME of ATV could be affected through either metabolic enzymes or transporters, merely detecting pharmacokinetics of orally administered ATV might mask drug-drug interactions that occur through mechanisms other than intestinal absorption. This study, therefore, investigated whether QSYQ altered liver Cyp3A1, Cyp3A2 and Oatp1b2 function, by analysing pharmacokinetics of ATV administered intravenously to avoid interference by transporters in the intestinal tract. Combined with pharmacokinetics of oral ATV, we are able to speculate about whether changes occurred in transporters on intestinal epithelial cells. The results from intravenously treated groups showed no significant difference in either ATV or *o*-ATV upon treatment with QSYQ. As mentioned above, *o*-ATV is the major metabolite produced by liver Cyp3A1 (Feidt et al. 2010; Zhang 2015), and the similarity of pharmacokinetics of *o*-ATV suggested that the transforming rate of ATV in liver was not influenced. Meanwhile, the pharmacokinetics of ATV did not change either, suggesting that absorption and elimination of ATV were similar in both intravenously treated groups. In orally treated rats, the pharmacokinetics of the major metabolite *o*-ATV were not significantly different, while the AUC_0-12 h_ and the *C_max_* of ATV increased by 80% and 95%, respectively. The *C_max_* of the minor metabolite *p*-ATV increased by 154%, but this increase was sufficiently small that it could be ignored, when comparing the AUC_0-12 h_ and the *C_max_* of *p*-ATV with *o*-ATV. Together, the results of analyses of orally and intravenously treated groups allow us to speculate that absorption of ATV is mainly influenced by QSYQ through transporters on intestinal epithelial cells.

The expression of metabolic enzymes and transporters tends to vary widely among different species, and the expression of the genes of metabolic enzymes and transporters vary widely in different organs within a species. For instance, the expression of P-glycoprotein gradually increases from duodenum to colon in rodents, the sites of high expression of Bcrp slightly different in rats and mice and the Oatp gene subtype in rat has few similarities to the OATP genes in human. Therefore, selecting the appropriate species and organ to study is critical in order to provide results that are minimally variable among individuals (Tanaka et al. 2005; Murakami and Takano 2008; Tamai 2012; Ulvestad et al. 2013). The study of MacLean et al. showed that in rats, from duodenum to colon, the level of expression of P-glycoprotein gradually increased, while the expression of Mrp2 gradually decreased, and the expression of Bcrp tends to be high in jejunum and ileum and low in duodenum and colon. In addition, the study revealed that Bcrp expression levels vary widely in the distal part of intestinal tract (i.e., the colon); on the contrary, Mrp2 expression levels most notably varied in the proximal part of the intestinal tract (i.e., the duodenum). In addition, this study showed that females have greater individual differences than males in the variation of Bcrp and Mrp2 (MacLean et al. 2008). Because this study suggested smaller variability of expression of transporters in the jejunum and ileum, we focussed our study on these sections of the intestinal tract.

The qRT-PCR results indicated that the target genes of P-glycoprotein and Mrp2 show similar variation in both orally and intravenously treated groups. The expression of the gene *Abcb1a*, which encodes P-glycoprotein, decreased significantly upon treatment with QSYQ in the jejunum of rats. Similarly, the expression of the gene *Abcc2,* which encodes Mrp2, decreased significantly in the ileum with QSYQ treatment. The protein abundance of P-glycoprotein in the duodenum and jejunum is lower than in the ileum and colon, which is opposite to Mrp2 (MacLean et al. 2008; Murakami and Takano 2008). QSYQ, then, might inhibit the expression of P-glycoprotein and Mrp2, especially in site where the abundance of the protein is already low.

The WB results suggested that expression of Mrp2 protein decreased significantly in ileum upon QSYQ treatment, while expression of P-glycoprotein was not significantly different in jejunum. There was no concordance between changes of P-glycoprotein mRNA and protein, and it is understandable because of a well-known phenomenon in which abundances of mRNAs are often poorly correlated with those of their proteins (Wang et al. 2013). Our results indicated that QSYQ treatment results in variation in P-glycoprotein and Mrp2 levels. AUC and *C_max_* levels increased due to the reduction of Mrp2 protein which allowed more ATV into the systemic circulation through the ileum.

In the process of drug absorption and distribution, the difference in the content of active ingredients of the drug in the intestinal lumen and blood might lead to inconsistent changes in Cyp3A1 and P-glycoprotein. A previous study investigated the serum levels of active ingredients achieved upon QSYQ treatment. Each gram of QSYQ dropping pills contains 14.52 mg tanshinol, 6.22 mg astragaloside IV, 9.83 mg ginsenoside Rg1 and 7.97 mg ginsenoside Rb1 (Fan et al. 2012). After intragastric administration of QSYQ at a dose of 6 g/kg, this study detected four major ingredients in rat blood, namely 22.23 μM tanshinol, 1.47 μM astragaloside IV, 8.34 μM ginsenoside Rg1 and 15.77 μM ginsenoside Rb1. These concentrations were lower than those achieved upon administration via gavage solution: 44.21 mM tanshinol, 4.50 mM astragaloside IV, 6.98 mM ginsenoside Rg1 and 4.15 mM ginsenoside Rb1. As tanshinol achieved the highest concentration in blood among the ingredients, it is a candidate for factor influencing the expression of CYP3A4. The mRNA expression level of *CYP3A4* has been shown to be significantly increased by 1 μM and 25 μM tanshinol (Liu et al. 2011). Interestingly, another study has shown that tanshinol was a weak inhibitor of CYP3A4 and the half inhibitory concentration was more than 200 μM (Qiu et al. 2008). According to these studies, tanshinol exhibited different effects on CYP3A4 at low and high concentrations. We speculate, then, that blood concentration of tanshinol in our study may have been, higher than 1 μM but lower than 25 μM and only increased the mRNA level of *Cyp3A1*. On the other hand, the gavage concentration of tanshinol, which is higher than 200 μM, decreased CYP3A4 expression.

In addition, 100 μM astragaloside IV can increase protein expression of P-glycoprotein without impacting the mRNA, while increasing the expression of *CYP3A4* mRNA with no effect on protein (Zhang et al. 2016). A blood concentration of astragaloside IV, which was lower than 100 μM, was shown to have no effect on Cyp3A1, while the gavage concentration of astragaloside IV, which was higher than 100 μM, increased P-glycoprotein expression. The phenomenon might offset the inhibitory effect of tanshinol on P-glycoprotein. The research on effects of different concentration of ginsenoside Rg1 and Rb1 on enzymes and transporters is not sufficient to allow the drawing of solid conclusions. When considering the concentrations of the four ingredients detected in the intestinal tract and blood, we believe that administering 675 mg/kg QSYQ to rats might alter mRNA or protein expression of P-glycoprotein owing to its high concentration in intestinal tract, but it might have no effect on Cyp3A1 due to low concentration in blood.

## Conclusion

QSYQ significantly increased ATV exposure by inhibiting the expression of Mrp2 in the ileum, which impacted groups treated orally but showed no effect on ATV pharmacokinetics in intravenously treated groups. The study revealed the influence of QSYQ on ADME of ATV by transporters in rats, but the sample size was limited. We only investigated the expression of transporters in jejunum and ileum; alterations in other sites need to be explored in the future. Results in rat models may not translate perfectly to humans due to different genotypes, but this report suggests that clinicians should pay close attention to adverse reactions, especially myopathy and rhabdomyolysis, when co-administering ATV and QSYQ.
